# Detection of Foot-and-Mouth Disease Virus in the Absence of Clinical Disease in Cattle and Buffalo in South East Asia

**DOI:** 10.3389/fvets.2021.691308

**Published:** 2021-07-23

**Authors:** Kelly Buckle, Rudolfo Bueno, Andrew McFadden, Mary van Andel, Richard Spence, Carolyn Hamill, Wendi Roe, Emilie Vallee, Fernanda Castillo-Alcala, Ronel Abila, Blesilda Verin, Bolortuya Purevsuren, Ashish Sutar, Htun Htun Win, Myo Thiha, Khin Ohnmar Lwin, Syseng Khounsy, Sengxay Phonthasy, Viliddeth Souriya, Chattouphone Keokhamphet, Jonathan Arzt, Anna Ludi, Valérie Mioulet

**Affiliations:** ^1^Ministry for Primary Industries, Biosecurity New Zealand, Diagnostic and Surveillance Services Directorate, Wallaceville, New Zealand; ^2^Ministry for Primary Industries, Office of the Chief Departmental Scientist, Wellington, New Zealand; ^3^School of Veterinary Science, Massey University, Palmerston North, New Zealand; ^4^OIE Sub-regional Representation for South East Asia, Bangkok, Thailand; ^5^Livestock Breeding and Veterinary Department, Nay Pyi Taw, Myanmar; ^6^Department of Livestock and Fisheries, Vientiane, Laos; ^7^Foreign Animal Disease Research Unit, Agricultural Research Service, United States Department of Agriculture, New York, NY, United States; ^8^The Pirbright Institute, Woking, United Kingdom

**Keywords:** abattoirs, surveillance, buffaloes, cattle, foot-and-mouth disease, foot-and-mouth disease virus, reverse transcriptase polymerase chain reaction

## Abstract

Foot-and-mouth disease virus (FMDV) is widespread throughout much of the world, including parts of South East Asia. Surveillance is often limited in endemic areas, relying predominantly on passive outbreak reporting. As part of the World Organisation for Animal Health (OIE)'s South East Asia and China Foot-and-Mouth Disease Project (SEACFMD), field sampling was performed to help understand evidence of widespread virus exposure observed in previous studies. Serum and dry mucosal swabs were collected to evaluate the presence of FMDV RNA on the nasal, oral, and dorsal nasopharyngeal mucosal surfaces of 262 healthy cattle (*n* = 84 in Laos; *n* = 125 in Myanmar) and buffalo (*n* = 48 in Laos; *n* = 5 in Myanmar) immediately following slaughter in three slaughterhouses. Swabs and serum were tested by the OIE/FAO World Reference Laboratory for foot-and-mouth disease (WRLFMD) using pan-serotypic real-time reverse transcription-PCR (rRT-PCR) and serum was evaluated using the FMD PrioCHECK non-structural protein (NSP) ELISA. In total, 7.3% of animals had detectable FMDV RNA in one or more of the three sites including 5.3% of nasopharyngeal swabs, 2.3% of oral swabs, and 1.5% of nasal swabs. No FMDV RNA was detected in serum. Overall, 37.8% of animals were positive for NSP antibodies, indicating likely past natural exposure to FMDV. Results were comparable for Laos and Myanmar, and for both cattle and buffalo, and were not significantly different between age groups. Detectable FMDV RNA present on the oral and nasal mucosa of clinically-healthy large ruminants in Laos and Myanmar demonstrates the importance of sampling asymptomatic animals as part of surveillance, and may indicate that subclinical infection plays a role in the epidemiology of FMD in these countries.

## Introduction

Foot-and-mouth disease virus (FMDV) is a contagious Picornavirus of cloven-hoofed ungulates (Artiodactyla), present in approximately two-thirds of the world's countries. In these countries, it acts as a significant barrier to trade (1, 2). Parameters of acute FMD infection in naïve cattle are well-defined, and continue to be refined by research (3, 4). In contrast, the subclinical cycles of FMD in the approximately 128 countries where the virus circulates are less-well documented, and the characteristic, “fulminant” herd-wide disease may be observed or reported inconsistently in endemic regions compared to what is documented in epidemic contexts (5, 6). Almost 50 years ago, Anderson et al. (7) wrote that “the occurrence of clinical outbreaks does not necessarily give a true assessment of the amount of virus in the environment as subclinical or in apparent infection could occur, particularly in partially immune cattle.” Much uncertainty remains about how FMD manifests in endemically-infected herds, and the various states by which subclinical infection exists (8).

The majority of naïve cattle exposed to FMDV will become infected through exposure of the mucosa of the upper respiratory tract, and will develop viraemia and lesions in a well-characterised pattern (9). Antibodies are a useful indicator of natural exposure, and may be partially cross-protective to future infection from other serovars (10). Cattle with viral infection past 28 days are defined as chronic carriers, with virus most commonly persisting in the nasopharyngeal mucosa (8, 11). In live chronic carriers, the probang technique, using a metal cup which is used to collect oropharyngeal fluid and mucosal cells from the pharyngeal region, provides the most useful sample of the area of viral persistence, allowing for molecular detection and virus isolation (8, 12). Scraping the same area with a cuvette at slaughter also yields virus (7).

The chronic carrier state is just one of several sub-clinical and immunological states of FMDV infection. It is a focus of research for FMD-free countries, largely because of its importance for declaration of freedom implications following an incursion. The other sub-clinical states of large ruminants are important for understanding the epidemiology of FMDV in endemic regions. These states include: (1) a pre-clinical (incubation phase) state between exposure and lesion development and (2) neoteric sub-clinical infection in which breed or species-associated host-adaptation precludes clinical signs of FMD (13, 14). Immunity and seropositivity may result from (1) vaccination with an appropriate serotype-specific vaccine and (2) an immune state resulting from previous exposure or maternal transfer of antibodies (strongest against the original infecting serotype).

Knowledge of in-country FMD epidemiology forms one of the major requirements for countries participating in the Food and Agriculture Organisation of the United Nations (FAO) and the World Organisation for Animal Health (OIE)'s Progressive Control Programme for FMD (PCP-FMD), which provides a benchmarking guide to countries wishing to progress toward FMD freedom (15). Protective natural immunity is not a factor addressed in FMD research, despite the high seroprevalence in some regions and the possible role this “herd immunity” plays in suppressing disease outbreaks in the face of circulating FMDV. In non-vaccinated populations of large ruminants, documented seroprevalence ranges from 19 to 71% in Africa (16–18), and between 18 and 51% in Asia (19–21).

Control of FMD has been identified as a priority for the livestock production sectors in Myanmar and Lao PDR, due to their perceived role in regional transmission of FMD due to cattle movement (22). Previous studies undertaken in these countries as part of the PCP-FMD demonstrated widespread exposure of cattle, with up to 56% of villages in central Myanmar demonstrating serological evidence of FMDV exposure (5), 51% of large ruminants at a Laos slaughterhouse testing positive on serology (20) and the circulation of multiple strains of FMDV (types O, A, and Asia 1) (23, 24). Seropositivity is greater than predicted by either formal outbreak reporting (25) or village headman observations (5). This suggests that research is needed to better define the role played by subclinical animals in FMDV transmission in Myanmar and other FMDV-endemic countries with similar husbandry and ecological conditions.

As part of addressing this research need, the present study evaluated whether FMDV is detectable on the mucosal surfaces of healthy cattle and buffalo in Laos and Myanmar, with samples taken at slaughter. The results of this study may help better understand the parameters of subclinical epidemiology of FMDV in the Southeast Asian region.

## Materials and Methods

**Figure 1:**
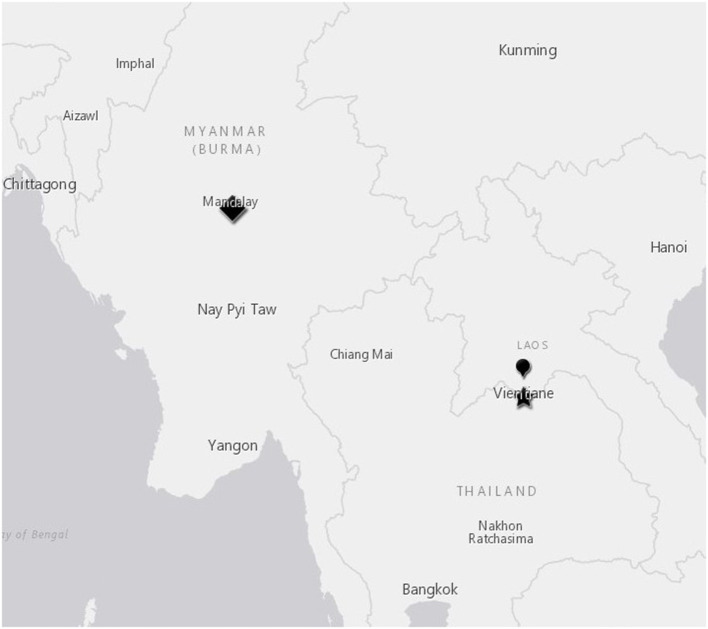
Slaughterhouse locations in Mandalay, Myanmar and Vientiane, Laos. Mandalay slaughterhouse, diamond symbol; Dongdou slaughterhouse, star symbol; Nangduang slaughterhouse, balloon symbol.

Slaughterhouse (SH) sites: sampling occurred at Dongdou SH and Nangduang SH (Vientiane, Laos) during May and June, 2019; and at Mandalay City SH, Mandalay, Myanmar during July and August, 2019 (Figure 1). Animal contact at the slaughterhouses varied—at Dongdou animals were gathered in a chute up to 12 h prior to slaughter, and secured to a railing with a headrope. At the other slaughterhouses, animals typically arrived in small groups by truck up to 24 h prior to slaughter and were tethered by a headrope to trucks or within the slaughterhouse, or kept in stalls with animals from one trader together until the time of slaughter. Animals from one trader could represent multiple original villages, districts or townships of origin. Animals were brought by traders to the slaughterhouse in groups, collected from different villages and transported together. For the Myanmar slaughterhouse, animals from 16 traders were sampled over five nights. In Laos, all animals tested at Dongdou were supplied by one trader, but at Nangduang (a more traditional slaughterhouse), animals were supplied by 10 traders over three nights.

Study population: Apparently healthy cattle and buffalo bound for human consumption were sampled opportunistically postmortem. Complete samples were obtained from 132 animals (84 cattle and 48 buffalo) in Laos, and 130 animals (125 cattle and 5 buffalo) in Myanmar. Animals were observed at a distance prior to slaughter, and no clinical disease was noted.

Samples: A sample set including serum and swabs from three sites (nasal, oral, and dorsal nasopharyngeal mucosae) were collected from each animal immediately after slaughter. Whole blood was collected in 10 mL red-top (plain) Vacutainer tubes (Becton, Dickinson and Company, Franklin Lakes, New Jersey, USA) following severing of the jugular vein as part of the normal slaughter process. Tubes were either centrifuged at 1,500 × *g* for 3 min, or left to clot within 12 h of collection. Serum (1 mL) was then collected into a 1.5 mL screw cap tube (Sarstedt) and frozen at −80°C until processing. Plain dry rayon Copan^TM^ swabs (Copan Diagnostics Inc., Murrieta, California USA) were collected from the above-mentioned sites during the slaughter process, were immediately inserted into a cryovial containing 1.0 mL of DNA/RNA Shield ^TM^ (Zymo Research), and were kept chilled on ice packs for between three to 12 h until arrival at the local laboratory where they were frozen at −80°C for transport to the World Reference Laboratory for FMD at the Pirbright Institute (Ash Road, Surrey, UK).

Swabs were collected in the following manner: Oral swabs were rubbed for approximately three seconds on the hard palate, buccal surfaces, and tongue as possible given the position of the animal, and were inserted up to the length of the 15 cm swab; nasal swabs were rubbed on all inside surfaces of both nostrils for 1–2 s each, with the swab inserted up to the length of the 15 cm swab into the nasal openings; pharyngeal samples prioritised sampling of the dorsal pharyngeal mucosa, the site of optimal experimental FMDV retrieval for persistently-infected animals (26) and involved insertion of the swab from a caudal direction through the oesophagus, with blind manual guidance to the dorsal nasopharyngeal mucosa, which was rubbed vigorously for 3–5 s. During initial sampling, dissection of a buffalo head confirmed that palpation of landmarks allowed sampling of the target mucosal surface. Where ruminal contamination was present, heads were pre-washed with water from a hose or bucket to minimise contamination of swabs. During collection, field staff employed frequent changing of gloves to prevent cross-contamination. Environmental control samples were collected each 10–15 carcasses by waving swabs through air adjacent to carcass collection sites (air controls) and by immersing swabs in local hose or trough water (water controls).

Data collected at sampling: Prior to slaughter, oral and nasal lesions and any signs of lameness were assessed and recorded. Animal species, age, and sex were collected from traders at the time of sampling. At the time of sampling, the external nares and rostral oral cavity were observed for the presence of gross lesions including vesicles, erosions or swellings.

Laboratory assays: The frozen serum and swab samples were maintained at −80°C at the National Animal Health Laboratory of the country of origin, then transported to Pirbright Institute on dry ice (Ash Road, Surrey, UK). Serum was evaluated for FMDV non-structural proteins (NSPs) using the FMD PrioCHECK NSP ELISA as per kit instructions with the exception that two wells were used per sample. Swabs were evaluated for the presence of FMDV RNA by the pan-serotypic 3D one-step real-time reverse transcription-PCR (RT-qPCR) (27). RT-PCR values were determined to be positive if the cycle threshold (CT) value was under 40.

Analysis: analyses were performed in R (R version 4.0.0 (2020-04-24) Copyright (C) 2020 The R Foundation for Statistical Computing) (28).

## Results

Detectable FMDV RNA was present on the oral and nasal mucosa of a small but consistent number of cattle and buffalo from both countries and all slaughterhouses. This included 3.4% (9/262, 95% CI 1.58–6.42%) of all animals which had detectable FMDV RNA on oral and/or nasal swabs. When pharyngeal swabs were added to the oral and nasal swabs, 7.3% (19/262, 95% CI 4.42–11.09%) of all animals in both countries had detectable FMDV RNA on at least one swab (Table 1), including 10.4% (5/48, 95% CI 3.47–22.66%) buffalo and 6.0% (5/84, 95% CI 1.96–13.34%) cattle in Laos, and 0% (0/5, 95% CI 0.00–52.18%) buffalo and 7.2% (9/125, 95% CI 3.34–13.23%) cattle in Myanmar. No animals had any lesions or clinical signs consistent with FMD. In the 19 animals with positive swabs, the most common site of detection was dorsal nasopharyngeal (14/19 animals), followed by oral (6/19 animals), and then nasal (4/19 animals). There were nine animals with positive oral and/or nasal swabs (9/262, 3.4%). Animals with more than one positive swab included four individuals, all cattle except where indicated: a 1-year old female positive on all swabs, a 2-year old female positive on both nasal and pharyngeal swabs, and a 3-year old female and 4-year old female buffalo with positive oral and pharyngeal swabs. Animals positive on at least one swab were significantly more likely to be seropositive (61.9% seropositive, 13/21) compared with animals from the whole group (101/262, 38.5%) (Chi-square statistic = 4.4084, *p* = 0.036). All environmental control swabs were negative by real time PCR.

**Table 1:** PCR and NSP ELISA results from healthy cattle and buffalo (*n* = 262) tested at slaughter in Laos (Dongdou and Nongduang) and Myanmar (Mandalay).

		**NSP ELISA Interp**	**NS PCR pos**	**OR PCR pos**	**DNP PCR pos**	**PCR pos any site**
Laos	Cattle	38/84 (45.2%)	4/84 (4.8%)	1/84 (1.2%)	3/84 (3.6%)	5/84 (6.0%)
	Buffalo	12/48 (25.0%)	0/48 (0%)	2/48 (4.2%)	4/48 (8.3%)	5/48 (10.4%)
	Total	50/132 (37.9%)	4/132 (3.0%)	3/132 (2.3%)	7/132 (5.3%)	10/132 (7.6%)
Myanmar	Cattle	47/125 (37.6%)	0/125 (0%)	3/125 (2.4%)	7/125 (5.6%)	9/125 (7.2%)
	Buffalo	2/5 (40.0%)	0/5 (0%)	0/5 (0%)	0/5 (0%)	0/5 (0%)
	Total	49/130 (37.7%)	0/130 (0%)	3/130 (2.3%)	7/130 (5.4%)	9/130 (6.9%)
Cattle total		85/209 (40.7%)	4/209 (1.9%)	4/209 (1.9%)	10/209 (4.8%)	14/209 (6.7%)
Buffalo total		14/53 (26.4%)	0/53 (0%)	2/53 (3.8%)	4/53 (7.5%)	5/53 (9.4%)
Total		99/262 (37.8%)	4/262 (1.5%)	6/262 (2.3%)	14/262 (5.3%)	19/262 (7.3%)

*Swab samples for RT-PCR were taken from three mucosal surfaces (oral, OR; nasal, NS; dorsal nasopharynx, DNP). Real-time PCR results were considered positive for detection of FMDV RNA at any CT value under 40*.

The study group was 81% female (212/262), and the rest were male except for one animal with unrecorded sex. Sex was not a significant factor in whether there were one or more positive swabs (1 positive male to 19 positive total or 5.3%, Fisher's exact test 95% CI (0.01–1.46), *p* = 0.137). The median age was 5 years old (median= 4yo for Laos, 5yo for Myanmar), and more young animals (under 24 months of age) were sampled in Laos (*n* = 37) compared to Myanmar (*n* = 4). The mean age of animals that were positive on one or more swab was 4.5 years, and age was not significantly different compared to animals that were negative on all swabs (Mann-Whitney *U* test, *W* = 2545.5, *p* = 0.4532), and no significant difference was found by grouping of animals into less than and greater-than 3 years old (Chi-square test, 1.7659, *p* = 0.1838). Animals with positive RT-PCR results were spread across multiple traders and across multiple nights.

## Discussion

In FMD-free countries, FMDV infection typically causes fulminant disease due to the widespread lack of immunity amongst host species. There is increasing evidence, including the data presented here, that FMDV infection in endemic regions can have more subtle manifestations including various forms of subclinical infection (21, 29, 30).

In the current study, we found that 3.4% of healthy, commercially slaughtered large ruminants from an endemic region had detectable FMDV RNA within the oral or nasal cavity. The results were similar for both Laos and Myanmar, suggesting this might be a pattern which is common throughout the region. This is supported by data from Myanmar showing that clinical FMD occurred less frequently than exposure and seroconversion (5). The FMDV 3D RT-qPCR has a diagnostic specificity of close to 100% (31) and a diagnostic sensitivity (DSe) of 97.7% (32), therefore low rates of false positivity may occur. Although false positivity could have altered the overall percent of true FMD RNA positives, this influence is likely to have been minimal. Virus isolation would be a useful next step in addressing false positivity, and should be considered in future studies.

To our knowledge, this is the first detection of FMDV by swab sampling on superficial mucosal surfaces of healthy cattle in South East Asia. Numerous studies have described subclinical detection of FMDV by probang sampling; however mucosal swab sampling has the advantage of not requiring specialised equipment and training. Previous work has shown inefficacy of FMDV detection by antemortem swabbing (33); however the current study demonstrates the utility of swab collection for surveillance in post-mortem settings. The origin of the FMDV detected is presumed to be the individual animals themselves, as no environmental contamination was detected and positive cases were scattered among locations and over several nights. Samples were taken immediately following slaughter, and site positivity was not consistently related, therefore cross-contamination between sites is assumed to have been minimal. In naïve cattle, oral and nasal shedding following experimental infection sharply declines over 21 days (34) with extinction of virus in the oral or nasal mucosa regardless of whether animals clear infection or become chronic carriers (11). The presence of FMDV RNA within the dorsal nasopharynx of chronic carriers is well-established (11, 12) among others. Our data suggest that small but consistent numbers of subclinical large ruminants could shed infectious FMDV particles from their oral and nasal mucosa. This may constitute an important, previously-unreported, mechanism of persistence and circulation of FMDV in endemic countries.

The animals sampled were chosen by convenience, and nothing was known about their previous exposure to FMDV except that they originated from an FMD-endemic region. None of the animals in this study had visible scars, lesions or other clinical signs (e.g., salivation, lameness) suggestive of current or recent FMD. We used NSP ELISA positivity as an imperfect proxy for prior exposure, as a way of judging whether presence of FMDV RNA might be related to pre-clinical acute (incubation phase) FMDV infection. Vaccination with whole vaccines (which could cause NSP-positive reactors) is thought to be low in both Laos and Myanmar, and was not considered likely to influence NSP ELISA results. In Laos, vaccination rates are thought to be as low as 18%, with incomplete adherence to vaccination schedules in many cases (35). Both countires in this study had recently carried out mass vaccination campaigns (36), however these utilised purified, subunit vaccines which would not have been detected by the serology assay used here. In our study, animals with detectable mucosal viral RNA at any site were significantly more likely to be seropositive than the sampled population, but not all RNA-positive animals had antibodies. Since development of antibodies postdates initial infection and early replication, animals with detectable FMDV RNA but no antibodies might have been in the incubation phase or the early (neoteric) stages of subclinical infection (8), or may have represented false negative reactors. The FMD PrioCHECK NSP ELISA has a diagnostic specificity of 99. 5% in vaccinated and 97.2% in non-vaccinated cattle, and a diagnostic sensitivity of 97.2% (37). There is some evidence that this sensitivity may further decrease in the face of natural infection. Three commercial NSP ELISAs were found to have a decreased diagnostic sensitivity of 21.6–28.4% when used on known-infected recovered cattle sera from the 2010 epidemic in Japan (38) compared to the previous findings (97.2% diagnostic sensitivity) by Brocchi et al. (37).

Six seropositive animals had detectable viral RNA in the oral and nasal cavity. Since NSP serology is not serotype specific, one possible reason for shedding is that these animals did not have protective antibodies to the same virus that was detected by RT-PCR. However, we consider it unlikely that all shedding animals were in the pre-clinical stages of FMD, and more likely (given the complete absence of lesions) that the presence of FMDV on mucosal surfaces constitutes a true subclinical state of FMD infection, either carrier phase or neoteric.

In the absence of herd or village level information on FMD clinical status, no inference can be made on a potential outbreak in the animals' original herd using group level data. Overrepresentation of disease is possible in slaughterhouse populations such as this one. For example, Vietnamese poultry smallholders were reported to increase their sales to mitigate losses associated with an outbreak (39). No lesions of any stage were observed during this study, and it is unlikely that all shedding cattle were in the pre-clinical stages of FMD. Therefore, we consider that these cattle are likely to have been sub-clinically infected with FMDV.

Outbreaks of FMD in Myanmar and Laos have been attributed to incursions of new strains of FMDV (23, 24). Our results suggest the possibility that in addition to these virus “incursions,” the endemic epidemiology of FMD could include subclinical infection and shedding within populations of apparently healthy animals. Experimental studies of viral location in chronic carrier cattle and buffalo focus heavily on nasopharyngeal virus detection (11, 21, 40). The observation that healthy dairy cattle and buffalo shed virus in their milk (41, 42), raises the possibility that in endemic countries, cattle could be exposed from birth to both virus and protective maternal antibodies.

Our study supports the well-established concept that FMDV is constantly circulating at low levels among fully or partially-resistant large ruminants in endemic regions of Southeast Asia. We used dry swabs for sampling rather than metal cuvettes used at slaughter by Anderson et al. (7); this was to prevent possible contamination between animals. One limitation of this study is that detection of FMD viral RNA is not proof of the presence of viable (infectious) FMDV. However, many studies have demonstrated the high correlation between detection of FMDV RNA and infectious virus from subclinical animals in endemic settings (6, 43). We suggest that future work includes confirmation of FMDV viability using virus isolation. Comparison of serotypes of FMDV and circulating antibodies might help to better understand the relationships between immunity and subclinical shedding. Furthermore, repeating this study in other regions and countries where FMDV is endemic, would help substantiate this missing piece of the FMD epidemiology puzzle in endemic regions. Sampling at the village of origin, rather than the slaughterhouse, could help to minimise mixing and the possibility of neoteric infection. With further work, the technique described here could potentially be adapted and utilised in other contexts, such as forming part of a surveillance program in countries with high vaccination coverage, wishing to monitor for subclinical shedding.

In summary, we found that 3.4% of healthy large ruminants at slaughter had detectable FMDV RNA in their oral and/or nasal cavities. Although the viability of this viral RNA was not examined in this study, our results suggest the possibility that FMDV present on the oral and nasal mucosa of asymptomatic large ruminants could play a yet-uncharacterised role in the epidemiology of FMD in Southeast Asia.

## Data Availability Statement

The raw data supporting the conclusions of this article will be made available by the authors, without undue reservation.

## Ethics Statement

Ethical review and approval was not required for the animal study because samples were obtained from dead animals at abattoirs.

## Author Contributions

KB, RB, AM, MA, RS, WR, RA, JA, and BV contributed to the conception and design of the study. KB, RB, BP, AS, CH, HW, MT, KL, SK, SP, VS, and CK contributed to logistics and operations of sample collection and fieldwork, including field-based alterations to the initial study design. AL and VM performed the laboratory analysis. KB wrote the first draft. AM, RB, JA, EV, and FC-A helped write drafts of the manuscript and assisted with analysis. All authors contributed to manuscript revision, read, and approved the submitted version.

## Conflict of Interest

The authors declare that the research was conducted in the absence of any commercial or financial relationships that could be construed as a potential conflict of interest.

## Publisher's Note

All claims expressed in this article are solely those of the authors and do not necessarily represent those of their affiliated organizations, or those of the publisher, the editors and the reviewers. Any product that may be evaluated in this article, or claim that may be made by its manufacturer, is not guaranteed or endorsed by the publisher.
